# Impact of occupational SARS-CoV-2 risk on the mental distress of non-healthcare employees in Germany: Swaantje Casjens

**DOI:** 10.1093/eurpub/ckac131.257

**Published:** 2022-10-25

**Authors:** S Casjens, D Taeger, T Brüning, T Behrens

**Affiliations:** Institute for Prevention and Occupational Medicine of the DGUV, Institute of the Ruhr University Bochum, Bochum, Germany

## Abstract

**Background:**

The SARS-CoV-2 pandemic posed major challenges to employees and companies. Differences between industries and professions in occupational SARS-CoV-2 infection risk (OSIR) became apparent early on. This study examines the mental distress in terms of depression and anxiety symptoms of non-healthcare workers during the first and second Corona waves in Germany.

**Methods:**

We conducted an online survey from December 2020 to June 2021 among employees from industrial enterprises, local public transport, the public and the financial sector. High and potential OSIR was defined based on job information. Depression and anxiety symptoms were rated with the 4-item Patient Health Questionnaire (PHQ-4). Categorized PHQ-4 scores were modeled with ordinal random-intercept logistic multinomial regression models and presented with odds ratios (OR) and 95% confidence intervals (95% CI).

**Results:**

Overall, 516 of 1,545 participants (33.4%) were determined to be at increased OSIR. Anxiety and depression symptoms worsened during the pandemic in all OSIR groups. Risks for more severe depressive and anxiety symptoms were higher among employees with high (OR 2.35; 95% CI 1.33-4.16) and potential OSIR (OR 1.70; 95% CI 1.19-2.43) compared to employees without OSIR. Severity of mental distress differed also by the extent of perceived job protection, interactions with colleagues, work-privacy conflicts, and overcommitment.

**Conclusions:**

OSIR had a negative impact on employee’s depressive symptoms and anxiety. Reducing SARS-CoV-2 exposure through workplace protective measures, strengthening interactions among colleagues, and supporting employees with work-privacy conflicts could help better protect employees’ mental health.

**Key messages:**

• The risk for more severe depressive and anxiety symptoms increases with higher occupational SARS-CoV-2 infection risk.

• Improvements in SARS-CoV-2 exposure at work, colleague interactions, and work-privacy conflicts could help better protect mental health.

